# Efficient Production of an Engineered Apoptin from Chicken Anemia Virus in a Recombinant *E. coli* for Tumor Therapeutic Applications

**DOI:** 10.1186/1472-6750-12-27

**Published:** 2012-06-06

**Authors:** Meng-Shiou Lee, Fang-Chun Sun, Chi-Hung Huang, Yi-Yang Lien, Shin-Huei Feng, Guan-Hua Lai, Meng-Shiunn Lee, Jung Chao, Hsi-Jien Chen, Jason T C Tzen, Hao-Yuan Cheng

**Affiliations:** 1 School of Chinese Pharmaceutical Sciences and Chinese Medicine Resources, China Medical University, Taichung, 40402, Taiwan, Republic of China; 2 Department of Bioresources, Da-Yeh University, Changhua, 51591, Taiwan, Republic of China; 3 Graduate School of Biotechnology, Hung Kuang University, Taichung, 43302, Taiwan, Republic of China; 4 Department of Veterinary Medicine, National Pingtung University of Science and Technology, Pingtung, 91201, Taiwan, Republic of China; 5 Graduate Institute of Biotechnology, College of Agriculture and Natural Resources, National Chung Hsing University, Taichung, Taiwan; 6 Department of Medical Research, Tung’s Taichung MetroHarbor Hospital, Taichung, 43344, Taiwan, Republic of China; 7 Institute of Pharmacology, National Yang-Ming University, College of Medicine, Taipei, 11221, Taiwan, Republic of China; 8 Department of Safety, Health and Environmental Engineering, Mingchi University of Technology, Taipei, 24301, Taiwan, Republic of China; 9 Department of Nursing, Chung Jen College of Nursing, Health Sciences and Management, Chia-Yi, Taiwan

## Abstract

**Background:**

Apoptin, a nonstructural protein encoded by the VP3 gene of chicken anemia virus (CAV), has been shown to not only induce apoptosis when introduced into the precursors of chicken thymocytes, but has been found to specifically kill human cancer cells, tumor cell and transformed cells without affecting the proliferation of normal cells. This tumor-specific apoptotic characteristic of the protein potentially may allow the development of a protein drug that has applications in tumor therapy. However, several major problems, which include poor expression and poor protein solubility, have hampered the production of apoptin in bacteria.

**Results:**

Significantly increased expression of recombinant full-length apoptin that originated from chicken anemia virus was demonstrated using an *E. coli* expression system. The CAV VP3 gene was fused with a synthetic sequence containing a trans-acting activator of transcription (TAT) protein transduction domain (PTD). The resulting construct was cloned into various different expression vectors and these were then expressed in various *E. coli* strains. The expression of the TAT-Apoptin in *E. coli* was significantly increased when TAT-Apoptin was fused with GST-tag rather than a His-tag. When the various rare amino acid codons of apoptin were optimized, the expression level of the GST**-**TAT-Apoptin_opt_ in *E. coli* BL21(DE3) was significantly further increased. The highest protein expression level obtained was 8.33 g/L per liter of bacterial culture after induction with 0.1 mM IPTG for 4 h at 25 °C. Moreover, approximately 90% of the expressed GST-TAT-Apoptin_opt_ under these conditions was soluble. After purification by GST affinity chromatography, the purified recombinant TAT-Apoptin_opt_ protein was used to evaluate the recombinant protein’s apoptotic activity on tumor cells. The results demonstrated that the *E. coli*-expressed GST-TAT-apoptin_opt_ showed apoptotic activity and was able to induce human premyelocytic leukemia HL-60 cells to enter apoptosis.

**Conclusions:**

On expression in *E. coli*, purified recombinant TAT-Apoptin_opt_ that has been fused to a GST tag and had its codons optimized, was found to have great potential. This protein may in the future allow the development of a therapeutic protein that is able to specifically kill tumor cells.

## Background

Chicken anemia virus (CAV), is a non-enveloped virus and the causative agent of chicken anemia disease. The disease results in aplastic anemia, lymph organ atrophy and immunosuppression in chickens [1-3]. The virus transcribes and translates three viral proteins, VP1, VP2 and VP3, which are respectively encoded by ORF3, ORF1 and ORF2 of the CAV genome. VP1 protein (51 kDa) is the sole structural protein of CAV and is responsible for capsid assembly [4]. The VP2 protein (30 kDa) is a nonstructural protein that possesses a dual-specificity protein phosphatase (DSP) [5]. The VP3 protein (13 kDa), also called apoptin, is a strong inducer of apoptosis in precursor chicken thymocytes and various human transformed and tumor cell lines but not in normal cells [6]. At present, the full anti-tumor mechanism of apoptin remains unclear. However, it is worth mentioning that apoptin-induced apoptosis in tumor cells is p53-independent and also is not suppressed by Bcl-2 or BCR-ABL protein [7]. Thus, apoptin is thought to be a good candidate for use as a therapeutic protein and has potential to be developed as a cancer treatment, including those cancers that lack p53.

To develop apoptin as an anti-cancer drug, efficient transduction tools such as recombinant virus and a recombinant plasmid within liposomes have been used to deliver apoptin into tumor cells [8]. However, these approaches have restrictions in terms of therapeutic application including a size limitation of the genes that can be delivered, insertional mutagenesis and transient expression [9-11]. Novel cell-penetrating peptides, such as the trans-acting activator of transcription (TAT) protein transduction domain (PTD), which consists of 11 amino acid residues (aa 47-57, YGRKKRRQRRR) have therefore been used as vectors for protein delivery [8,12]. With respect to apoptin, previously studies have been demonstrated that apoptin fused with a TAT peptide shows apoptotic activity against tumor cells because the TAT peptide’s delivery system is able to move apoptin into cells [13,14].

Up to the present, a number of different expression systems have been used to express apoptin, including *E. coli*, baculovirus-insect cells and plant cells [15-18]. However, production of recombinant full-length apoptin/VP3 protein has generally been possible only in *E. coli*[17]. Several production problems involving the expression efficiency and protein solubility of apoptin in *E. coli* have been encountered [13,17,18]. Thus, there is a need to overcome these difficulties in order to scale-up production of full-length apoptin protein using an *E. coli* expression system. If successful, this would not only allow the efficient development of a therapeutic protein that is able to actively kill cancer cells specifically but the recombinant protein would also be potentially useful when developing diagnostic kits for the clinical detection of CAV infection [17].

It is clear that *E. coli* has a number of limitations and disadvantages in terms of the production of apoptin protein. However, expression of apoptin protein in *E. coli* is still an attractive alternative to the current production system when assessed in terms of cost, time and operational considerations. In this study, the CAV VP3 gene was fused to a synthetic gene containing the trans-acting activator of transcription (TAT) protein transduction domain (PTD). This protein, named TAT-Apoptin, was expressed using various different expression vectors in order to evaluate TAT-Apoptin expression and production by a number of different *E. coli* strains. Two expression vectors were used, one harboring a glutathione-S-transferase (GST) tag and the other a 6xHis tag; these were investigated to explore the effect of these fusion tags on the expression of TAT-Apoptin in the various *E. coli* strains. In addition, changes in codon usage for various amino acids within the VP3 gene were also assessed in terms of their effect on expression of TAT-Apoptin_opt_. Rare codons for *E. coli* within the VP3 gene of the TAT-Apoptin protein were optimized using prediction software and the preferred codon usage changed to that of *E. coli*. After this optimization, the expression levels of the modified VP3 genes were examined in various *E. coli* strains and the various production parameters for TAT-Apoptin_opt_ protein expression assessed. To the best of our knowledge, the yield of *E. coli* expressed recombinant TAT-Apoptin_opt_ in this study after codon optimization of the VP3 gene is the highest known to date.

## Results

### A GST fusion tag improves the expression of recombinant TAT-Apoptin protein in *E. coli*

**Figure 1:**
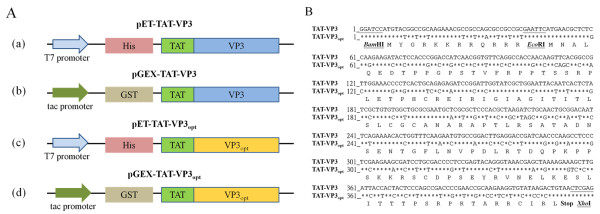
**Schematic diagram of the constructs used for TAT-Apoptin protein expression.** (**A**) Schematic representation of the TAT-Apoptin protein fused with different affinity tags together with the expression vectors used in this study. The designations of the TAT-Apoptin protein and its expression vectors are indicated, (**a**), (**b**), (**c**) and (**d**). The constructs, (**a**) and (**b**), contain the full-length TAT-VP3 gene cloned into the vectors pET28a and pGEX-4 T-1; these were used for expression of TAT-Apoptin protein with either a six-histidine (6 × His) tag or a glutathione-s-transferase (GST) tag at the N-terminus, respectively. Constructs (**c**) and (**d**) containing the TAT-VP3 gene that was codon-optimized; this was derived from construct (**b**) by replacing rare codons without altering the amino acid sequence. The codon-optimized TAT-VP3gene, TAT-VP3_opt_, was then cloned into pET28a and pGEX-4 T-1. (**B**) Sequence comparison between the TAT-VP3 gene and the TAT-VP3_opt_ gene. The nucleotide sequences were compared between the original TAT-VP3 gene (wild type TAT-VP3) and the sequence of codon-optimized TAT-VP3 gene (TAT-VP3_Opt_) over the whole coding region. An asterisk (*) represents the fact that the aligned nucleotides are identical.

**Figure 2:**
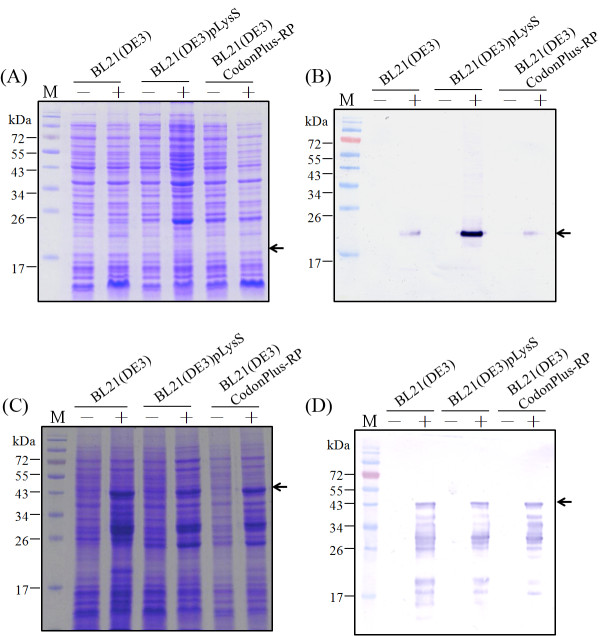
**Expression of recombinant TAT-Apoptin protein in three different*E. coli*strains.** The TAT-Apoptin protein expression in three *E. coli* strains, BL21(DE3), BL21(DE3)pLysS and BL21(DE3)CodonPlus-RP, which were transformed with either pET-TAT-VP3 or pGEX-TAT-VP3 and cultivated at 37 °C. His-TAT-Apoptin and GST-TAT-Apoptin protein were examined and detected using SDS-PAGE (**A, C**) and Western-blotting (**B, D**). Anti-His and anti-GST tag monoclonal antibodies was respectively used to recognize the His-TAT-Apoptin protein and GST-TAT-Apoptin. Lane M, pre-stained protein marker; the symbols “-” and “+” represented pre-induction and post-induction with 1 mM of IPTG over 4 hrs of cultivation in *E. coli*, respectively.

**Figure 3:**
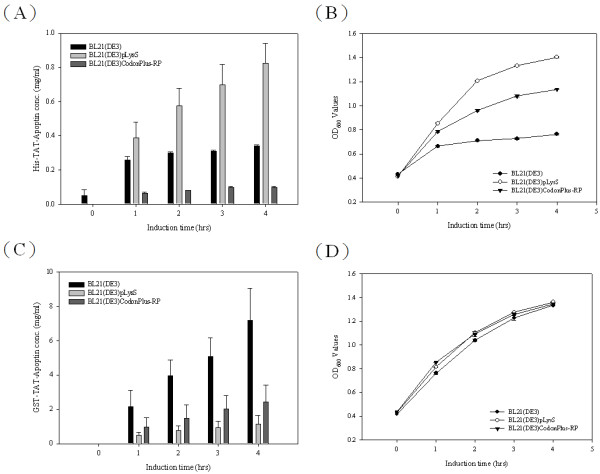
**Productivities of TAT-Apoptin protein and the growth curves of the three recombinant*E. coli*strains.** The productivities of His-TAT-Apoptin (**A**) and GST-TAT-Apoptin (**C**) for the three *E. coli* strains, BL21(DE3), BL21(DE3)pLysS, and BL21(DE3)CodonPlus-RP containing pET-TAT-VP3 or pGEX-TAT-VP3 are shown over the time course of cultivation at 37 °C after IPTG induction. The growth curves of BL21(DE3), BL21(DE3)CodonPlus-RP and BL21(DE3)pLysS expressing His-TAT-Apoptin (**B**) and GST-TAT-Apoptin (**D**), respectively, in LB medium post-induction.

To develop apoptin protein as anti-tumor drug, a composite cDNA of the CAV VP3 gene fused synthetic gene of TAT peptide was used to create two distinct expression constructs of TAT-Apoptin (Figure 1A, a and b). These constructs, pET-TAT-VP3 and pGEX-TAT-VP3, were transformed into three different *E. coli* strains in order to investigate the effect of the fusion tags on the expression of TAT-Apoptin. The 6 × His and GST tags were fused with TAT-Apoptin at the N-terminus using the expression vectors pET28a and pGEX-4 T-1, respectively. The expression of these two TAT-Apoptin constructs in various *E. coli* strains was explored (Figure 2). When *E. coli* BL21(DE3) was used, significant amounts of full-length TAT-Apoptin protein were present in the whole cell lysate after IPTG induction for 4 h with either pET-TAT-VP3 or pGEX-TAT-VP3 (Figure 2B, SDS-PAGE and Western-blotting). The 16 kDa His-TAT-Apoptin and the 42 kDa GST-TAT-Apoptin were detected using monoclonal anti-His antibody and anti-GST antibody, respectively (Figure 2B, lane 4 of Western-blot). Densitometric analysis of the blots showed that total expressed GST-TAT-Apoptin protein, both soluble and insoluble, from BL21(DE3) was approximately 19 fold greater than that of His-TAT-Apoptin produced under identical conditions (Figure 3A and C). When His-TAT-Apoptin and GST-TAT-Apoptin were induced for between 1 and 4 h, the expression of both proteins continued to increase significantly over the 2-4 h induction range (Figure 3A and C) with the highest amount of His-TAT-Apoptin protein produced being 0.34 mg/ml at 4 h of IPTG induction. In contrast, the highest amount of GST-TAT-Apoptin produced was 7.13 mg/ml at 4 h. A similar pattern of expression was observed for the two proteins in BL21(DE3)CodonPlus-RP and BL21(DE3)pLysS strains (Figure 2). The highest amounts of GST-TAT-Apoptin produced by BL21(DE3)CodonPlus-RP and by BL21(DE3)pLysS were 2.43 and 1.15 mg/ml, respectively, after 4 h of IPTG induction (Figure 3A and C). The parallel results for His-TAT-Apoptin were 0.09 and 0.82 mg/ml, respectively (Figure 3A and C). Thus the GST fusion tag improved protein expression significantly and allowed large amounts of intact TAT-Apoptin protein to be produced.

### VP3 gene encoding apoptin protein of CAV is rich in *E. coli* rare codons

CAV VP3 gene that encodes the apoptin protein consists of 121 amino acid codons. The rare codons for *E. coli* within the VP3 gene were pinpointed using the GenScript Rare Codon Analysis Tool (http://www.genscript.com/cgi-bin/tools/rare_codon_analysis). The deduced amino acid sequence of apoptin was found to contain 15% basic amino acid residues such as arginine (R) and lysine (K) (Figure 1B). Overall, approximately 21% rare *E. coli* codons were present in the VP3 gene, including arginine, leucine, isoleucine, proline, cysteine, threonine, serine and glycine codons (Figure 1B). The presence of these codons is one possible reason for the relatively poor expression of apoptin in *E. coli*.

### Enhancement of recombinant TAT-Apoptin protein expression in *E. coli* by optimizing the codon usage of the VP3 gene

**Figure 4:**
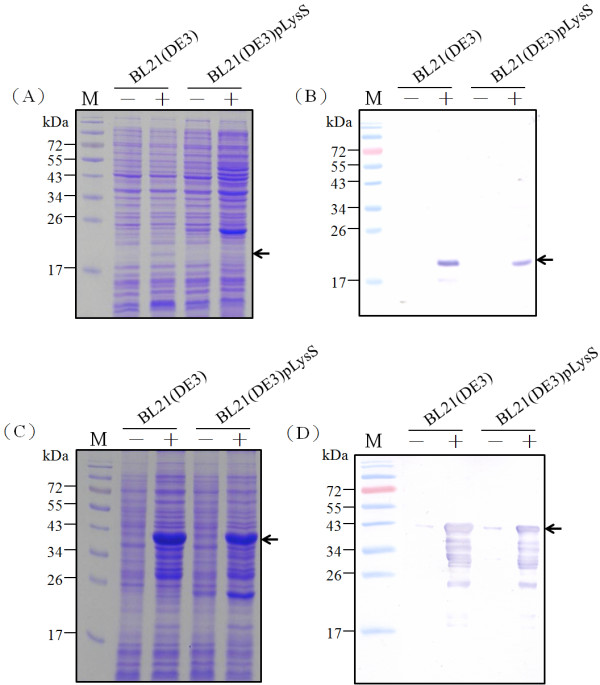
**Expression of recombinant TAT-Apoptin**_**opt**_**protein in the different*E. coli*strains.** The TAT-Apoptin_opt_ protein was expressed in the *E. coli* strains BL21(DE3) and BL21(DE3)pLysS, which contained either pET-TAT-VP3_opt_ or pGEX-TAT-VP3_opt_, at 37 °C. His-TAT-Apoptin_opt_ and GST-TAT-Apoptin_opt_ protein were examined and detected using SDS-PAGE (**A**, **C**) and Western-blotting (**B**, **D**). Anti-His and anti-GST tag monoclonal antibodies was respectively used to recognize the His-TAT-Apoptin_opt_ protein and GST-TAT-Apoptin_opt_. Lane M, pre-stained protein marker; “-” and “+” represented pre-induction and post-induction with 1 mM of IPTG over 4 hrs of cultivation in *E. coli*, respectively.

**Figure 5:**
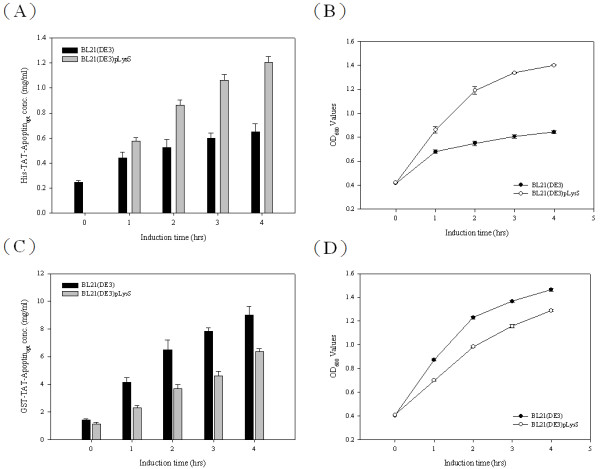
**Productivities of TAT-Apoptin**_**opt**_**protein and the growth curves of two recombinant*E. coli*strains.** The productivities of His-TAT-Apoptin_opt_ (**A**) and GST-TAT-Apoptin_opt_ (**C**) using two *E. coli* strains, BL21(DE3) and BL21(DE3)pLysS containing either pET-TAT-VP3_opt_ or pGEX-TAT-VP3_opt_, respectively, are shown over a time course after IPTG induction at 37 °C. The growth curves of BL21(DE3) and BL21(DE3)pLysS expressing His-TAT-Apoptin_opt_ (**B**) and GST-TAT-Apoptin_opt_ (**D**) in LB medium post-induction.

We have shown that expression of full-length TAT-Apoptin in *E. coli* is improved by fusing a GST tag to the N-terminus of the TAT-Apoptin protein. However, adding a His tag to give His-TAT-Apoptin did not produce such a yield improvement even when the optimal host strain BL21(DE3)pLysS is used (Figures 2A and 3A). Therefore, we next explored the effect on TAT-Apoptin productivity of optimizing the codon usage of the VP3 gene for *E. coli*. The TAT-Apoptin gene was engineered such that AGA/CGA/CGG were changed to CGT or CGC (R), CCC/CCT were changed to CCG or CCA (P), CTC/CTA/TTG were changed to CTG (L), ATA was changed to ATC (I), GGA/GGG were changed to GGT (G), ACT/ACA were changed to ACC (T), and CAA was changed to CAG (Q) (Figure 1B). The codon optimized VP3 gene was then linked to the TAT sequence at 5’ end of the VP3 gene to give an intact open reading frame. The new codon-optimized TAT-VP3 gene, denoted TAT-VP3_opt_, was then cloned into pGEX-4 T-1 and pET28a to give pGEX-TAT-VP3_opt_ and pET-TAT-VP3_opt_, respectively (Figure 1A, d and c). These plasmids were then individually expressed in *E*. coli. When expression in *E. coli* strain BL21(DE3) using whole cell lysate was analyzed by SDS-PAGE and Western blotting, both GST-TAT-Apoptin_opt_ and His-TAT-Apoptin_opt_ were successfully expressed after IPTG induction (Figure 4A and C) with a yield after 4 h IPTG induction of about 9 mg/ml and 0.65 mg/ml, respectively. The yield of GST-TAT-Apoptin_opt_ was almost 13 fold higher than His-TAT-Apoptin_opt_ (0.65 mg/ ml) (Figure 5A and C). BL21(DE3)-pLysS, similarly, gave increased expression of both His- TAT-Apoptin_opt_ and GST-TAT-Apoptin_opt_ (Figure 4A and C). The highest yields of GST-TAT-Apoptin_opt_ and His-TAT-Apoptin_opt_ in BL21(DE3)pLysS were obtained after 4 h IPTG induction and were 6.38 and 1.2 mg/ml, respectively (Figure 5A and C). These results confirm that the codon-optimized of TAT-Apoptin improved protein expression significantly and allowed large amounts of intact TAT-Apoptin_opt_ protein to be produced in either *E. coli* BL21(DE3) or BL21(DE3)pLysS with either fusion tag.

### Effect of cultivation temperature and IPTG concentration on the protein solubility of *E. coli*-expressed GST-TAT-Apoptin_opt_

**Figure 6:**
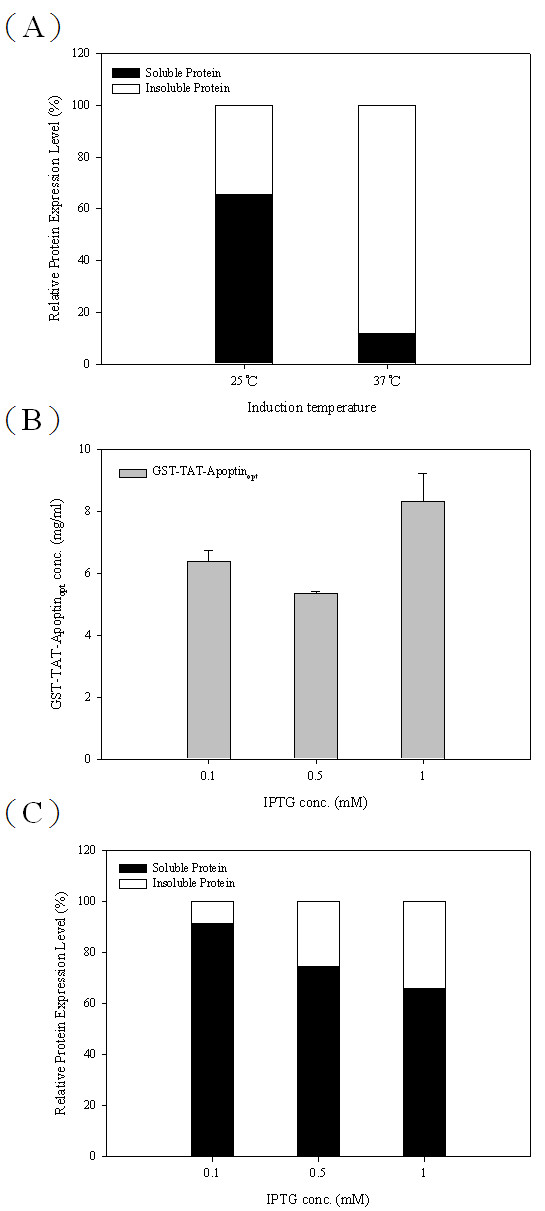
**Solubility of*E. coli*-expressed GST-TAT-Apoptin**_**opt**_**protein under using various cultivation parameters during protein induction.** The solubility of GST-TAT-Apoptin_opt_ was determined in the BL21(DE3) strain at different cultivation temperatures (**A**) and in the presence of various concentrations of IPTG (**B**, **C**).

**Figure 7:**
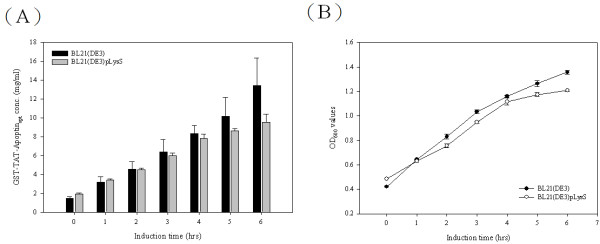
**Productivities of TAT-Apoptin**_**opt**_**protein and the growth curves in two recombinant*E. coli*strains.** The productivities of GST-TAT-Apoptin_opt_ using two *E. coli* strains, BL21(DE3) and BL21(DE3)pLysS containing pGEX-TAT-VP3_opt_ are shown over time after IPTG induction at 25 °C (**A**). Growth curves of BL21(DE3) and BL21(DE3)pLysS expressing GST-TAT-Apoptin_opt_, respectively, in LB medium post-induction (**B**).

In addition to the expression level of TAT-Apoptin_opt_, the solubility of TAT-Apoptin_opt_ protein is also needs to examine and improved. To this end, the cultivation parameters and their effect on the TAT-Apoptin_opt_ protein solubility were explored, namely cultivation temperature and the IPTG concentration for induction; GST-TAT-Apoptin_opt_ was used as the model protein. Protein solubility and the expression levels of GST-TAT-Apoptin_opt_ were determined using *E. coli* BL21 (DE3) at 25 °C and 37 °C. As shown in Figure 6A, an increased amount of soluble GST-TAT-Apoptin_opt_ was obtained after IPTG induction at 25 °C. Approximately 15% and 65% of the GST-TAT-Apoptin_opt_ in the whole cell lysate of the BL21(DE3) strain was soluble at 37 °C and 25 °C, respectively. In addition, the amounts of GST-TAT-Apoptin_opt_ protein expressed in BL21(DE3) were 8.33 mg/ml and 8.99 mg/ml, respectively, at 25 °C and 37 °C after 4 h IPTG induction (Figures 5C and 7A); thus the total amount of GST-TAT-Apoptin_opt_ protein obtained at 25 °C compared to 37 °C was only very slightly lower (Figures 5C and 7A).

When various IPTG induction concentration from 0.1 to 1 mM were explored for protein induction, the highest amount of GST-TAT-Apoptin_opt_ protein obtained was with 1 mM IPTG induction using the BL21(DE3) strain (Figure 6B) and this gave about 65% soluble protein at 25 °C (Figure 6C). The expression level of GST-TAT-Apoptin_opt_ at 0.1 and 0.5 mM IPTG was lower than for 1 mM IPTG (Figure 6B), but the amount of soluble protein was highest at 0.1 mM (95%) IPTG (Figure 6C). Thus a lower cultivation temperature of 25 °C and an IPTG concentration of 0.1 mM IPTG were able to produce the higher amount of soluble GST-TAT-Apoptin_opt_ protein.

### Purification of recombinant GST-TAT-Apoptin_opt_ protein using GST affinity chromatography

**Figure 8:**
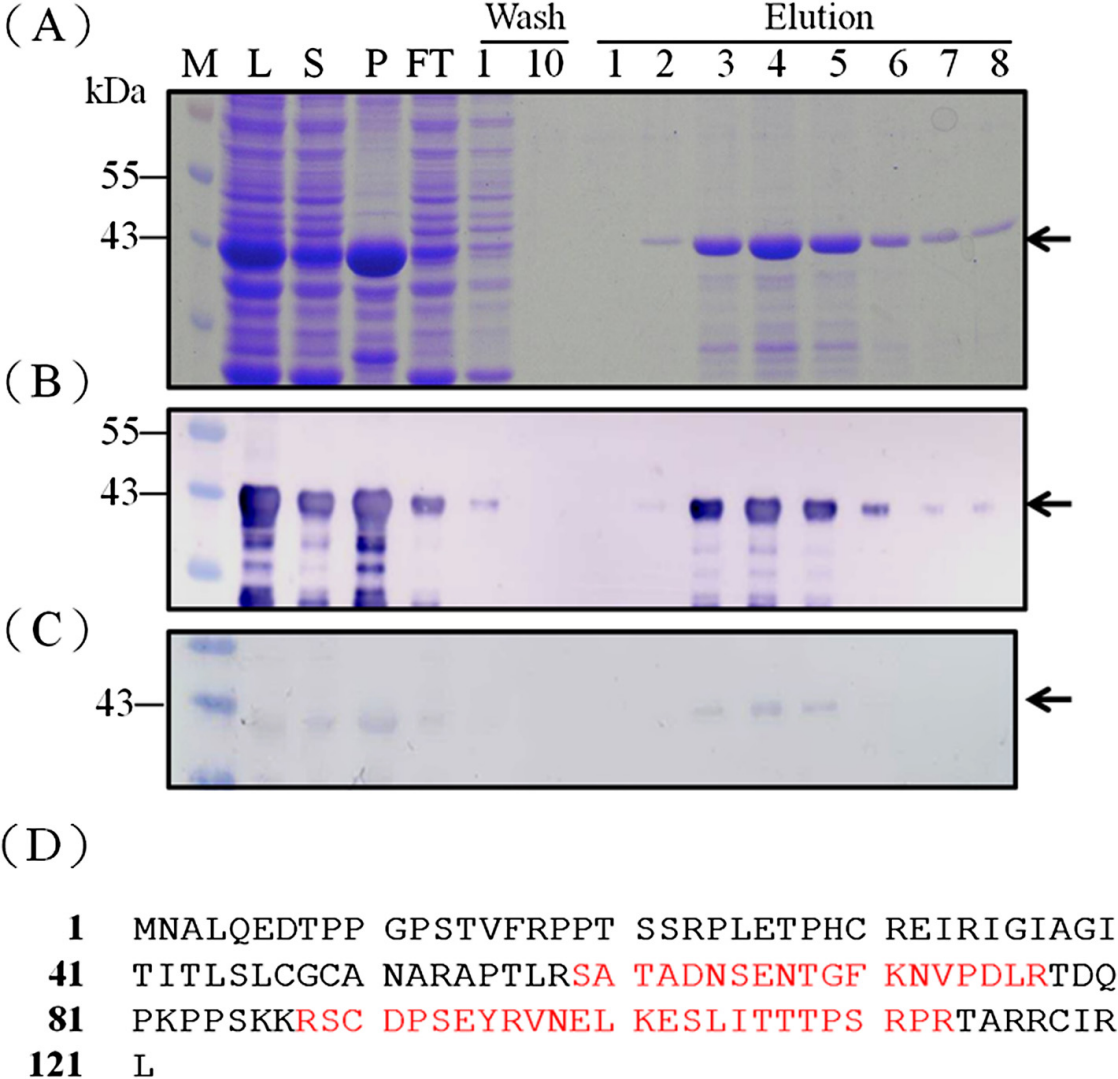
**Purification of recombinant GST-TAT-Apoptin**_**opt**_**protein.** SDS-PAGE(**A**) and Western-blot (**B**) analysis of the GST-TAT-Apoptin_opt_ protein contained in various elution fractions collected from the GSTrap FF affinity column. The cytosolic extract of *E. coli* strain BL21(DE3) expressing GST-TAT-Apoptin_opt_ protein was loaded onto a GSTrap FF column and the bound protein was eluted with elution buffer as described in Material and Methods. The eluted protein from the GSTrap FF affinity column was analyzed by SDS-PAGE and Western blotting using monoclonal anti-GST antibody. (**C**) Antigenicity analysis of GST-TAT-Apoptin_opt_. The purified GST-TAT-Apoptin_opt_ was assayed by Western blotting using positive CAV-infected chicken serum. Lane M, pre-stained protein marker; lane 1, flow through; lane 2, fraction obtained after column washing, lane 3 and 4, eluted fraction 1 and 2, respectively, collecting after column elution.(**D**) Identity of the GST-opt-VP1 protein determined by MALDI-TOF. The bold letters represent actual amino acid matches to published amino acid sequence (Accession No. AF212490).

Using the approximately 95% soluble GST-TAT-Apoptin_opt_ expressed by *E. coli* at 25 °C and 0.1 mM IPTG, the lysate was subjected to purification using a GST affinity column. After affinity chromatography, the eluted soluble GST-TAT-Apoptin_opt_ protein was confirmed antigenically using anti-GST antibody and CAV-infected positive serum (Figure 8A, B and C). A typical elution profile of the protein fractions collected from a GST column is shown in Figure 8A after separation by SDS-PAGE. Fraction 4 contains the most eluted protein, has a significant absorbent peak at OD_280_ and was eluted at 12 min (data not shown). The specific 42 kDa band eluted in fraction 4 was almost purified to homogenicity (Figure 8A, lane 4 of elution). When the purified GST-TAT-Apoptin_opt_ protein was examined by MALDI-TOF, seven peptides from GST-TAT-Apoptin_opt_ could be identified after trypsin digestion and these demonstrated good alignment and a high score when compared to the predicted protein (data not shown). The longest peptide fragment, VNELKESLITTTPSRPR, consists of 17 amino acid residues and overall the coverage was 35.7% of the published amino acid sequence of apoptin (Accession No. AF212490) without any miss-match (Figure 8D). These MALDI-TOF results confirmed that the purified 42 kDa protein is GST-TAT-Apoptin_opt_ and that the *E. coli* preferred codon usage optimization within the VP3 gene has not altered either the amino acid sequence (Figure 8C; Figure 8B, lane 4) or the antigenicity (Figure 8C) of the protein.

### Recombinant GST-TAT-Apoptin_opt_ protein has apoptotic activity and induces apoptosis in HL-60

**Figure 9:**
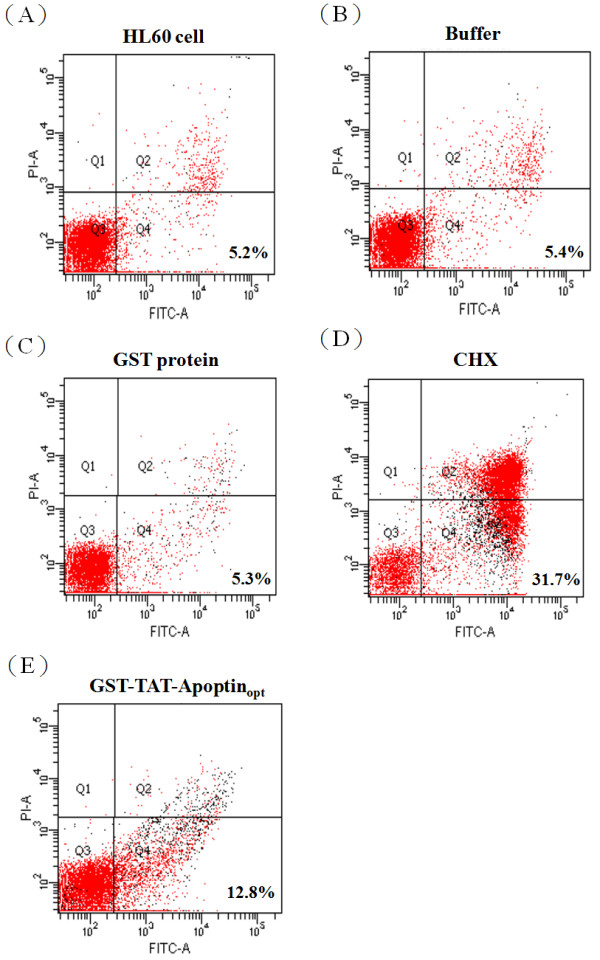
**Induction of apoptosis in HL-60 cells by GST-TAT-Apoptin**_**opt**_**.** An apoptotic assay of HL-60 cells was performed by flow cytometry after 90 ug/ml of the purified *E. coli*-expressed recombinant GST-TAT-Apoptin_opt_ protein was co-cultured with HL-60 cell for 24 hours (**D**). Non-apoptosis controls, (**A**), (**B**) and (**C**), were assayed. These represent respectively HL-60 cells only, HL-60 cells co-cultured with protein buffer and HL-60 cells co-cultured with GST protein. The apoptosis induced by CHX was used as a positive apoptosis control.

To investigate whether GST-TAT-Apoptin_opt_ protein expressed by *E. coli* has apoptotic activity when introduced into tumor cells, purified GST-TAT-Apoptin_opt_ protein was used to examine the protein's apoptotic activity when it was used to treat human premyelocytic leukemia HL-60 cells. As illustrated in Figure 9, flow cytometry analysis by Annexin-V FITC and propidium iodide staining showed an approximately 10% increase in the level of apoptotic levels among HL-60 cells that were co-cultured with GST-TAT-Apoptin_opt_ compared to HL-60 cells only, HL-60 co-cultured with GST or chromatographic elution buffer. However, compared to the control, HL-60 cells co-cultured with GST-TAT-Apoptin_opt_ induced less apoptosis than when HL-60 cells were induced into apoptosis by treatment with cyclohexamide (CHX). Nevertheless, GST-TAT-Apoptin_opt_ was able to induce apoptosis in HL-60 cells to a meaningful degree. These results indicate that *E.coli* expressed GST-TAT-Apoptin_opt_ retains the protein’s original apoptotic activity when used to treated HL-60 cells even after the modifications carried out in this study.

## Discussion

Apoptin from chicken anemia virus has been demonstrated to have apoptotic activity and to be able to specifically kill several types of tumor cells including the cell lines HeLa, Saos-2, lung cancer cells H1299 and HepG2 [9-11,13,14,19]. Apoptin is not only p53 and Bcl^-^ independent, but also does not require specific post-modification in order to be able to induce apoptosis [7]. Therefore, apoptin has great potential to be developed into a protein drug that will be useful as part of the cancer therapy armory. In this context, previously studies have shown that fusing the TAT peptide to apoptin, in order to create a recombinant protein vehicle, which allows protein uptake by cells, improves protein translocation into the cell and increases Saos-2 cell killing [19]. This approach is a useful was of overcoming problems associated with the delivery of apoptin into cells without affecting the anti-cancer activity of the protein. However, up to the present, few studies have investigated anti-cancer activity using such a recombinant TAT-Apoptin protein. The main reason for this lack of progress is the poor expression and low protein solubility of TAT-Apoptin. Previous studies have shown that the large-scale production of recombinant TAT-Apoptin using a prokaryotic expression is difficult and therefore this has become a bottle-neck [17,18]. Even today, in spite of the fact that many eukaryotic expression systems are well established, various factors, such as cost-effectiveness, insertional mutagenesis and transient expression, still need to be resolved for these systems. Taking the above into consideration, the effective production of TAT-Apoptin protein using a prokaryotic system is a crucial key step in developing this protein drug as an anti-tumor therapy. Therefore, in this study, the specific aim was to develop a prokaryotic expression system that allowed the efficient production of recombinant TAT-Apoptin protein. Using a prokaryotic expression system to express heterologous recombinant protein has several advantages including time-savings, cost-effectiveness, ease of production, simplified characterization and others. It is for these reasons that *E. coli* is the most used expression system when evaluating the expression of a foreign protein [20]. Indeed, the above points are some of the critical factors associated with choosing a suitable production system when developing a protein drug. To this end, problems associated with having a truly effective system for the large-scale production of apoptin protein still needed to be explored.

To improve protein expression and to enhance the solubility of any protein produced in an *E. coli* expression system, a number of strategies are available. These include cultivation parameters, the effect of fusing the protein to an affinity tag and the optimization of codon usage of foreign gene for *E. coli*. All of these have been frequently employed to improve the amount of recombinant protein recovered [17,18,21-23]. In the present study, we first explored the effect of two different fusion tags on TAT-Apoptin expression, although others remain available, are untested as yet and may further improve the yield in the future. The presence of a GST fusion tag was found to significantly improve the yield of TAT-Apoptin compared to a 6 × His tag (Figures 2 and 3). In a previous study, Liu *et al* successfully overcame the problem of less efficient expression in *E. coli* of porcine circovirus (PCV) by fusing the maltose-binding protein (MBP) to an 8xHis tag [23]. The main mechanism by which the MBP-8xHis tag improved protein expression remains unclear. However, one possibility is improved protein solubility [24]. Similarly, in our previous study, the addition of a GST tag to the CAV VP1 protein also improved expression in *E. coli* significantly compared to a His × 6 tag [21]. Thus it would seem that some fusion tags are able to improve expression of soluble protein in *E. coli* compared to other tags, perhaps by aiding the correct folding of their fused partner [21,24].

Next we investigated which of three different *E. coli* strains, BL21(DE3), BL21(DE3)pLysS and BL21(DE3)Codonplus-RP, was able to improve protein production and yield. With both GST-TAT-Apoptin and His-TAT-Apoptin, BL21(DE3) was preferred and produced more TAT-Apoptin protein than either BL21(DE3)pLysS or BL21(DE3)Codonplus-RP (Figure 2A and C). It is worth noting that BL21(DE3)pLysS has a higher growth rate than BL21(DE3) or BL21(DE3)Codonplus-RP when expressing His-TAT-Apoptin (Figure 2B and D). This discrepancy may involve either poor protein stability or the cytotoxic nature of His-TAT-Apoptin when present in BL21(DE3) and BL21(DE3)Codonplus-RP. These results are similar to those obtained for the production of GST-VP1 protein from CAV in *E. coli* at low expression levels [21]. BL21(DE3)pLysS superiority may be due to the presence in the strain of the *pLysS* plasmid during protein induction [17,21]. This difference may enable BL21(DE3)pLysS to tolerate cytotoxicity associated with the expression of T7 lysozyme by attenuating the transcription leakage by T7 RNA polymerase [17,21]. However, this phenomenon was not important when GST-TAT-Apoptin was expressed and the growth profiles of the three strains producing GST-TAT-Apoptin were almost identical on induction by IPTG. In this area, it is possible that the cytotoxicity of GST-TAT-Apoptin may be less that that of His-TAT-Apoptin in BL21(DE3) and BL21(DE3)Codonplus-RP [21]. Furthermore, the phenomenon of “protein burden” may not have been encountered when BL21(DE3) was used to express GST-TAT-Apoptin [25]. It was concluded that BL21(DE3) is the preferred choice for expression of GST-TAT-Apoptin.

Rosenberg *et al* have proposed that the abundance of a rare codon near the 5’-end of the gene might affect the efficiency of protein translation [26]. If this is true, then the approach used in the present study to modify the codon usage in the gene ought to improve the expression of full-length apoptin protein. When Genscript OptimumGene^TM^ bioinformatic software was used to identify rare codons in *E. coli* that exist in the wild-type CAV VP3 gene, the pinpointed amino acid residues included arginine, leucine, proline and lysine; these are commonly found in the N-terminus and C-terminus regions of the apoptin protein. After codon optimization of CAV VP3 gene, TAT-Apoptin_opt_ was fused with both tags and successful expressed in *E. coli* (Figure 4A, C). The amount of expressed GST-TAT-Apoptin_opt_ in *E. coli* was substantially higher than that of GST-TAT-Apoptin and His-TAT-Apoptin_opt_ when BL21(DE3) or BL21(DE3)pLysS were used (Figure 5A, C and Table 1). Thus codon optimization within VP3 gene of the rare codons in *E. coli* was able to improve the expression level of TAT-Apoptin; specifically translation efficiency was improved without the need to supply extra copies of the rare tRNA genes [27,28]. Interestingly, in terms of growth, BL21(DE3) and BL21(DE3)pLysS performed almost identically when expressing GST-TAT-Apoptin (Figure 3D). However, the growth rate of BL21(DE3)pLysS was significantly slower than that of BL21(DE3) when expressing GST-TAT-Apoptin_opt_ (Figure 5D). This might be explained in terms of the “protein burden” within BL21(DE3)pLysS when it is producing GST-TAT-Apoptin_opt_ at a relatively high level early during induction and this protein burden may eventually result in BL21(DE3)pLysS undergoing growth arrest. The growth profile of BL21(DE3) expressing GST-TAT-Apoptin_opt_ may involve the balancing of the steady-state growth conditions for the strain with the yield and also suggests that this strain had reached the maximum possible growth rate; as a result the growth profile of BL21(DE3) during the expression of GST-TAT-Apoptin_opt_ did not significantly change (Table 1, Figures 3D and 5D). In contrast, when producing His-TAT-Apoptin_opt_, the growth profiles of BL21(DE3) and BL21(DE3)pLysS were very similar to the situation when the strains were producing His-TAT-Apoptin (Figures 3B and 5B). One possible explanation for this is that the level of produced His-TAT-Apoptin or His-TAT-Apoptin_opt_ did not reach the threshold value where there was a protein burden and therefore no growth arrest occurred.

**Table 1:** **Summary of the productivities of the various TAT-Apoptin proteins expressed in the range of*E. coli*strains**

**Protein**	**Productivity of*E. coli*strains (mg/ml)**			**Plasmid**
	**BL21(DE3)**	**pLysS**	**CodonPlus-RP**	
**His-TAT-Apoptin**	**0.34 ± 0.01**	**0.82 ± 0.11**	**0.09 ± 0.01**	**pET-TAT-VP3**
**GST-TAT-Apoptin**	**7.13 ± 1.88**	**1.15 ± 0.49**	**2.43 ± 0.98**	**pGEX-TAT-VP3**
**His-TAT-Apoptin**_**opt**_	**0.65 ± 0.06**	**1.20 ± 0.05**	**-**	**pET-TAT-VP3**_**opt**_
**GST-TAT-Apoptin**_**opt**_^**a**^	**8.99 ± 0.65**	**6.38 ± 0.18**	**-**	**pGEX-TAT-VP3**_**opt**_
**GST-TAT-Apoptin**_**opt**_^**b**^	**8.33 ± 0.89**	**7.84 ± 0.44**	**-**	**pGEX-TAT-VP3**_**opt**_

^**a**^***E. coli*Strains were cultured at 37 °C.**

^**b**^***E. coli*Strains were cultured at 25 °C.**

The presence of a fusion tag and optimization of the codon usage within the gene were both useful strategies for improving the production of TAT-Apoptin (Table 1). When compared, the increase level in yield obtained when a fusion tag was used seems to be greater than that derived from codon optimization (Table 1). Specifically, the yield was only increased from 7.1 mg/ml (GST-TAT-Apoptin) to 8.9 mg/ml (GST-TAT-Apoptin_opt_) was expressed in BL21(DE3).

In order to improve the protein stability of TAT-Apoptin, various cultivation parameters were adjusted. Although a cultivation temperature of 37 °C was able to produce a higher growth rate and greater protein yield, this temperature did not produce a high yield of soluble protein. When 25 °C was used or when a lower IPTG concentration was used (0.1 mM of IPTG), it was not only possible to obtain a similar expression level of TAT-Apoptin within 6 hrs (compared to 4 hrs), but there was also a significant improvement in protein solubility (Figures 6A, 7A). Recovering soluble TAT-Apoptin using the GST tag as an affinity ligand during the purification process is highly convenient in terms of downstream processing. After GST affinity chromatography, approximately 80% of the GST-TAT-Apoptin produced by the *E. coli* was recovered and recovery of GST-TAT-Apoptin even reached over 90% with one batch (Figure 8). The purified target protein can then be subjected to further purification steps in order to obtain a protein drug with high purity.

In this study, human premyelocytic leukemia HL-60 cells were used to evaluate the apoptotic activity of the *E. coli*-expressed TAT-Apoptin. Apoptosis was induced when the HL-60 cells were co-cultured with 90 ug/ml of GST column purified GST-TAT-Apoptin_opt_. There was a 10% increase in apoptosis compared to the non-apoptosis control (Figure 9A, B and C). However, this is less apoptosis than that induced by CHX, which is the positive apoptosis control. Previous studies have reported that approximately 70% of tumor cells undergoing apoptosis in the presence of apoptin [11]. This discrepancy may be a result of differences in the tumor cell lines used, which may have different levels of tolerance with respect to apoptin. In addition, the TAT-Apoptin carried the GST fusion tag had not undergo protease cleavage before use and the presence of the tag may have affected the protein drug's apoptotic activity during co-culture with the tumor cells. Nevertheless, it is clear that the *E. coli*-expressed GST-TAT-Apoptin_opt_ produced in the present study does retain its anti-tumor cell activity and the ability to induce apoptosis.

## Conclusions

The expression of recombinant full-length TAT-Apoptin protein was established successfully herein using a prokaryotic system, and the yield was significantly improved by fusing the protein with an affinity tag and by optimizing the codon usage of the polypeptide for expression in *E. coli*. Direct engineering of the apoptin protein was a convenient and cost-effective strategy of increasing the expression of TAT-Apoptin protein in *E. coli*. This paves the way for the large-scale production of TAT-Apoptin protein using this approach. In the future, this will also allow TAT-Apoptin to be used for the development of a protein drug that has anti-cancer activity or as part of a CAV diagnostic test.

## Methods

### Bacterial strain and cells inoculation

Three *E. coli* strains, BL21(DE3) (Invitrogen, Carlsbad, CA), BL21(DE3)CodonPlus-RP (Stratagene, La Jolla, CA) and BL21(DE3)pLysS (Stratagene, La Jolla, CA) were used and maintained at 37 °C using 10 ml Luria-Bertani (LB)medium (1% tryptone, 0.5% yeast extract, 1% NaCl, pH 7.0) in 50 ml flasks. For strain activation, 0.5 ml of an overnight culture were inoculated into 50 ml LB medium and grown at 37 °C for around 3 h, by which time the optical density of culture had reach 0.5 of OD_600_. These bacterial cells could then be used for transformation or for protein expression.

### Construction of the recombinant plasmids

A 420 bp of synthetic cDNA encoding the full-length CAV apoptin protein that was fused to a trans-acting activator of transcription (TAT) protein transduction domain (PTD) at its N-terminus, the latter being synthesized by Genemark Biosci & Tech Co. (Taichung, Taiwan) and then ligated into pBluescript II SK(−) using *Bam* H1 (Takara, Japan) and *Xho* I (Takara, Japan) restriction sites. The resulting recombinant plasmid was designated pB-TAT-VP3. The *TAT-VP3* gene then was subcloned into either pET28a (Novagen, Madison, WI) or pGEX-4 T-1 (GE Healthcare, Piscataway, NJ) using *Bam* H1 and *Xho* I. The resulting constructs were designated pET-TAT-VP3 and pGEX-TAT-VP3 (Figure 1A, a and b), respectively. To generate the *VP3* gene of CAV harboring the codon optimized nucleotide sequence, a codon optimized fragment of *VP3* gene fused with the *TAT* PDT was also synthesized by Genemark Biosci & Tech Co. and then ligated into the pBluescript II SK(−) using *Bam* H1 and *Xho* I restriction sites. The *TAT-VP3*_opt_ gene was then further subcloned into either pET28a or pGEX-4 T-1 as described above and the resulting constructs were designated pET-TAT-VP3 _opt_ and pGEX-TAT-VP3_opt_, respectively (Figure 1A, c and d). The constructs described above were then transformed into One Shot® Top10 (Invitrogen, CA) chemically competent *E. coli* for maintenance of the recombinant plasmids and for protein expression. Transformants that contained a gene of the correct size by PCR were then confirmed as correct by restriction enzyme digestion and by DNA sequence analysis.

### Expression of TAT-Apoptin protein and codon optimized TAT-Apoptin_opt_ protein in recombinant *E. coli*

To express the His-TAT-Apoptin, GST-TAT-Apoptin, His-TAT-Apoptin_opt_ and GST-TAT-Apoptin_opt_ proteins, the four constructed recombinant plasmids, pET-TAT-VP3, pGEX-TAT-Apoptin, pET-TAT-VP3_opt_ and pGEX-TAT-VP3_opt_, were transformed into *E. coli* strain to allow evaluation of protein expression. Three *E. coli* host strains, BL21(DE3), BL21(DE3)CodonPlus-RP and BL21 (DE3)pLysS, each being a different recombinant constructions, were used for protein induction and expression. The recombinant strains were grown in LB medium in the presence of kanamycin (50 μg/ml), ampicillin (50 μg/ml) or chloramphenicol (34 μg/ml) as appropriate at 37 °C. When the culture had reached an optical density (OD_600_) of 0.5, isopropyl-β-D-thiogalactopyronoside (IPTG) at different concentrations was added to induce protein expression and then growth was continued for 4 h. After IPTG induction, samples of the cells were harvested and analyzed for protein expression. Protein concentration was determined using the procedure described in a previous study [21]. Samples containing the various expressed proteins, His-TAT-Apoptin or His-TAT-Apoptin_opt_ and GST-TAT-Apoptin or GST-TAT-Apoptin_opt_, were analyzed by 12.5% SDS-PAGE and Western-blotting using a monoclonal anti-His antibody (Invitrogen, Carlsbad, CA) or a monoclonal anti-GST antibody (GE healthcare, Piscataway, NJ).

### Purification of recombinant GST-TAT-Apoptin_opt_ protein using GST affinity chromatography

To purify the recombinant GST-TAT-Apoptin_opt_ proteins, cells were spun down from 50 mL of culture supernatant and resuspended in GST resin binding buffer (140 mM NaCl, 2.7 mM KCl, 10 mM Na_2_HPO_4_, 1.8 mM KH_2_PO_4_, pH 7.3). The mixture was then sonicated on ice three times for 3 minutes with a 20% pulsed activity cycle (MISONIX Sonicator® 300). Next, the lysate was centrifuged for 10 min at 10,000 rpm to remove the cell debris. The resulting cell supernatant was loaded onto a GSTrap FF affinity column (GE healthcare, Piscataway, NJ) for protein purification using the standard procedure described in a previous study [21]. The total protein concentration of each collected fraction from the column was determined using a Micro BCA kit (Pierce, Rockford, IL) with bovine serum albumin acting as the reference protein. The purity of the protein from each fraction was analyzed by 12.5% SDS-PAGE and then the resulting gels were Western blotted using monoclonal anti-GST antibody (GE healthcare, Piscataway, NJ).

### Mass spectrometry

To confirm the identity of the recombinant apoptin protein, *E. coli* expressed GST-TAT-Apoptin was purified by GSTrap FF column, and then the eluted proteins containing in collected fraction were separated by 12.5% SDS-PAGE. The relevant band was then cut out from the 12.5% SDS-PAGE gel after Coomassie blue staining and digested with trypsin. The resulting samples were subjected to the MALDI-TOF-MS mass spectrometry (ESI-QUAD-TOF) to allow amino acid sequence identification of the protein, as described in a previous study [17].

### Apoptosis assay

Human promyelocytic leukemia HL-60 cells (CCRC 60043) were purchased from the Food Industry Research and Development Institute (FIRDI) (Hsichu. Taiwan). These cells were used to evaluate the apoptotic activity of GST-TAT-Apoptin_opt_ using an Annexin-V FITC apoptosis kit (BD Phaemingen, San Diego, CA). The cell was grown in the FIRDI-suggested medium, and all experiments were performed in 6-well culture plates. The cultured HL-60 cells were stained with Annexin-V FITC and propidium iodide (PI) according to manufacturer’s guidelines. Briefly, 2 × 10^5^ cells were co-cultured with 90 ug/ml of GST-TAT-Apoptin_opt_ for 24 hrs, and then resuspended in 100 ul of 1× binding buffer, which was followed by incubation with 2.5 ul of Annexin-V FITC and PI at room temperature for 30 min. Finally the cells were added to 200 ul of 1× binding buffer and assayed immediately using FACSCanto flow cytometry (BD Biosciences, San Jose, CA). A positive control was included and this consisted of treating the cells with 100 mM cycloheximide (CHX) (Sigma-Aldrich, USA) for 24 hrs. The negative controls were either cells treated with protein buffer (50 mM Tris-HCl, 10 mM reduced glutathione, pH 8.0) or cells treated with 90 ug/ml glutathione-S-transferase (GST) protein for 24 hrs.

## Competing interests

All of authors declare no competing interests.

## Authors’ contributions

MSL participated in this study design, performed the experiments and helped with the writing of the manuscript. SHF, GHL and FCS performed the experiments and participated in the construction of the plasmids. YYL participated in the experiments on protein antigenicity and GHL, CHH and JC participated in the protein purification. HJC and MSL^6^ participated in the data analysis and the writing of the manuscript. MSL, HYC and JTCT coordinated the study and participated in the writing of the manuscript. All authors read and approved the final manuscript.
